# Medical versus surgical treatment of first trimester spontaneous abortion: A cost-minimization analysis

**DOI:** 10.1371/journal.pone.0210449

**Published:** 2019-01-10

**Authors:** Ana Cubo Nava, Zandra M. Soto Pino, Ana M. Haro Pérez, M. Estrella Hernández Hernández, M. José Doyague Sánchez, José M. Sayagués Manzano

**Affiliations:** 1 Departament of Obstetrics and Gynecology, Complejo Asistencial Universitario de Salamanca, Salamanca, Spain; 2 Department of Preventive Medicine and Public Health, Complejo Asistencial Universitario de Salamanca, Salamanca, Spain; 3 Departament of Hematology, Complejo Asistencial Universitario de Salamanca, Salamanca, Spain; 4 Institute for Biomedical Research (IBSAL), Salamanca, Spain; University of Insubria, ITALY

## Abstract

**Background:**

Traditionally the gold-standard technique for the treatment of spontaneous abortion has been uterine evacuation by aspiration curettage. However, many studies have proposed medical treatment with misoprostol as an alternative to the conventional surgical treatment. The aim of this study was to apply cost minimization methods to compare the cost and effectiveness of the use of vaginal misoprostol as a medical treatment for first trimester spontaneous abortion with those of evacuation curettage as a surgical treatment.

**Methodology/Principal findings:**

We present a longitudinal, prospective and quasi-experimental research study including a total of 547 patients diagnosed with first-trimester spontaneous abortion, in the period from January 2013 to December 2015. Patients were offered medical treatment with 800 mg vaginal misoprostol or evacuation curettage. Patients treated with misoprostol were followed-up at 7 days and a transvaginal ultrasound was performed to confirm the success of the treatment. If it failed, a second dose of 800 mg of vaginal misoprostol was prescribed and a new control ultrasound was performed. In case of failure of medical treatment after the second dose of misoprostol, evacuation curettage was indicated. The effectiveness of each of the treatment options was calculated using a decision tree. The cost minimization study was carried out by weighting each cost according to the effectiveness of each branch of the treatment. Of the 547 patients who participated in the study, 348 (64%) chose medical treatment and 199 (36%) chose surgical treatment. The overall effectiveness of medical treatment was 81% (283/348) and surgical treatment of 100%. The estimated final cost for medical treatment was € 461.92 compared to € 2038.72 for surgical treatment, which represents an estimated average saving per patient of € 1576.8.

**Conclusions/Significance:**

Medical treatment with misoprostol is a cheaper alternative to surgery: in the Spanish Public Healthcare System, it is five times more inexpensive than curettage. Given its success rates higher than 80%, mild side effects, controllable with additional medication and the high degree of overall satisfaction, it should be prioritized over the evacuation curettage in patients who meet the treatment criteria.

## Introduction

Clinical spontaneous abortion complicates 8–20% of pregnancies, of which 80% occur before 12 weeks of gestation [1–2]. Traditionally, the gold-standard technique for the treatment of spontaneous abortion has been uterine evacuation by aspiration curettage. However, several studies have proposed medical treatment with misoprostol as an alternative to conventional surgical treatment [3–5]. In 2005 Zhang et al.[6] published a randomized clinical trial demonstrating misoprostol to be an acceptable alternative to surgical treatment for the management of early pregnancy loss in terms of effectiveness, safety, acceptability and adverse effects. Other studies have confirmed the efficacy and safety of the medical treatment of early abortion compared with the classic surgical approach in selected cases [7]. This evidence has prompted the progressive modification of the protocols so that medical treatment with misoprostol is now regarded as being the primary option for the management of early gestational loss in most of the hospitals in Spain.

The medical treatment of first-trimester spontaneous abortion has several advantages over a surgical treatment: it can be provided in an outpatient regimen; it does not require hospital admission or anaesthesia; and, *a priori*, it seems to be a more cost-effective treatment than surgical treatment. However, very few published studies have attempted to quantify the magnitude of the possible savings associated with this new treatment strategy and some of their results are contradictory [8–11].

This study aimed to assess the effectiveness of vaginal misoprostol as a medical treatment for first-trimester spontaneous abortion versus evacuation-curettage as a surgical treatment and, if both were equivalent, to quantify the difference in costs of each procedure through a cost-minimization study.

## Material and methods

This is a longitudinal, prospective and quasi-experimental research study. A total of 547 patients diagnosed with first-trimester spontaneous abortion during the period between January 2013 and December 2015 in the Obstetrics and Gynaecology Unit of the University Hospital of Salamanca (Salamanca, Spain) were recruited after obtaining their informed consent. Patients who meet the inclusion criteria were offered both treatment options, and received medical treatment (n = 348, 64%) or surgical treatment (n = 199, 36%) according to patient’s choice. The misoprostol treatment protocol and the informed consents were evaluated and accepted by the Ethics Committee of the University Hospital of Salamanca. Data files are publicly available in Harvard Dataverse [12].

### Sample size calculation

The sample size was calculated using the Epi-Info program [13], based on the real number of deliveries in our hospital and considering the 20% risk of early pregnancy loss previously described by other studies [1–2]. For an estimated population of 6,300 deliveries, with an expected abortion rate of 20%, a 5% accuracy and a 95% confidence level, a total sample of 236 patients was estimated to be needed to conduct the study.

### Inclusion and exclusion criteria

The study included patients older than 18 years, with a single pregnancy with embryonic or fetal death, incomplete abortion with endometrium ≥16 mm or anembryonic pregnancy with a gestational sac of up to 45 mm confirmed by ultrasound, following the criteria previously described by Zhang et al. [6] but, since all patients were managed as outpatients, those with a fetus with a fetal crown-rump length (CRL) > 30 mm were not included because of the risk of heavy bleeding at expulsion of the pregnancy at home.

The rest of the inclusion criteria were: no signs of haemodynamic instability, haemoglobin values >9 g/dL, without known coagulopathy or absolute contraindications for the use of prostaglandins (glaucoma, severe asthma, mitral insufficiency and stenosis, and adrenal insufficiency, among others), and no history of allergy or hypersensitivity to misoprostol. Patients who did not meet these criteria or who declined to participate in the study were excluded.

The criterion for successful medical treatment was the diagnosis of complete abortion, established by an endometrial thickness ≤15 mm. Medical treatment was considered to have failed if, after the first or second dose of treatment, it was not possible to achieve complete uterine evacuation and a new curettage was needed to complete the process.

After the diagnosis of spontaneous abortion, blood tests were done to assess the haemoglobin level and the Rh-antigen status. A physical examination was also performed. If inclusion criteria were met, both treatment strategies were explained, and the patient herself chose the therapeutic option. The dosage used for the medical treatment was that proposed by the Spanish Society of Gynaecology and Obstetrics (SEGO) [2]: 800 μg of misoprostol administered vaginally, repeated after 7 days if the patient opted for a second dose of the medical treatment. No third dose was offered. Thus, if the patient opted for medical treatment, she was supplied with four 200-μg tablets of misoprostol to be self-administered vaginally in the patient’s own home. Patient was advised about the warning signs indicating the need of going to the Accident and Emergency Department and provided with analgesic medication to be used when required (paracetamol 500 mg, 3 tablets; ketorolac-tromethamine 25 mg, 3 tablets). A new appointment was made for a week after starting the treatment to assess by ultrasound whether the first dose had been successful. If the treatment was successful, the patient was discharged, but if it had failed, the options to repeat the medical treatment or to undergo a surgical treatment were offered again. If the patient decided to repeat the medical treatment, four more 200 μg misoprostol tablets were given for vaginal self-administration at home and a further appointment was arranged for 7 days later. If this second dose was successful, the woman was discharged. If it failed, surgical treatment was indicated.

If the patient chose surgical treatment as a first option, she was admitted to hospital, 400 μg of misoprostol were placed vaginally and, after 6 hours, an operating room vacuum aspiration was performed under anaesthetic sedation. The average time of admission was 24 hours.

In both cases, the patient signed informed consent to the chosen option. In the case of the medical treatment, a fact sheet was also provided explaining the possible complications and warning signs.

### Effectiveness calculation

The effectiveness of each branch of treatment was calculated by constructing a decision tree reflecting all the possible combinations of success and failure for the medical and surgical treatment options. The individual effectiveness of each alternative was taken to be the percentage frequency of that alternative with respect to its group (medical or surgical). The overall effectiveness of each treatment branch was obtained by adding the individual frequencies of each alternative for the medical or surgical treatment option.

### Costs calculation

The costs of each resource were provided by the Billing Department of the University Hospital of Salamanca (Salamanca, Spain). The costs of medical treatment were calculated as the sum of the costs of the various resources used. The cost of surgical treatment is set out in the Diagnosis Related Groups (DRG 381) of the National Health System (Sistema Nacional de Salud, SNS), under *"Abortion with dilation and curettage*, *aspiration or hysterotomy"*. In 2015, the overall cost was estimated to be €2019.37 [14].

### Economic evaluation

To carry out the economic evaluation, a cost-minimization method was performed [15,16]. A decision tree containing every possible option within the study was made, and the estimated cost for each specific procedure was assigned. The cost of each option was multiplied by its calculated effectiveness. The overall costs of the medical and surgical treatment options were estimated by adding the costs of each component procedure multiplied by its effectiveness or success percentage [15,16].

### Statistical methods

This study was conceived as a non-inferiority study, following the model of Zhang et al, which assumes that medical treatment of abortion would not be more effective than surgical treatment [6]. As in the aforementioned study, a threshold of 80% effectiveness in the medical treatment group was considered to be equivalent to that of surgical treatment.

The mean, standard deviation and 95% confidence interval of all continuous variables were calculated using SPSS v23 (IBM SPSS, Armonk, NY, USA). To establish the statistical significance of between-group differences of continuous variables, Student’s unpaired samples T test and the Mann-Whitney U test were used, depending on whether they were normally or non-normally distributed, respectively. The Pearson χ^2^ test was used to examine proportional differences between cross-tabulated qualitative variables. In all cases, statistical significance was concluded for values of p<0.05.

## Results

**Fig 1:**
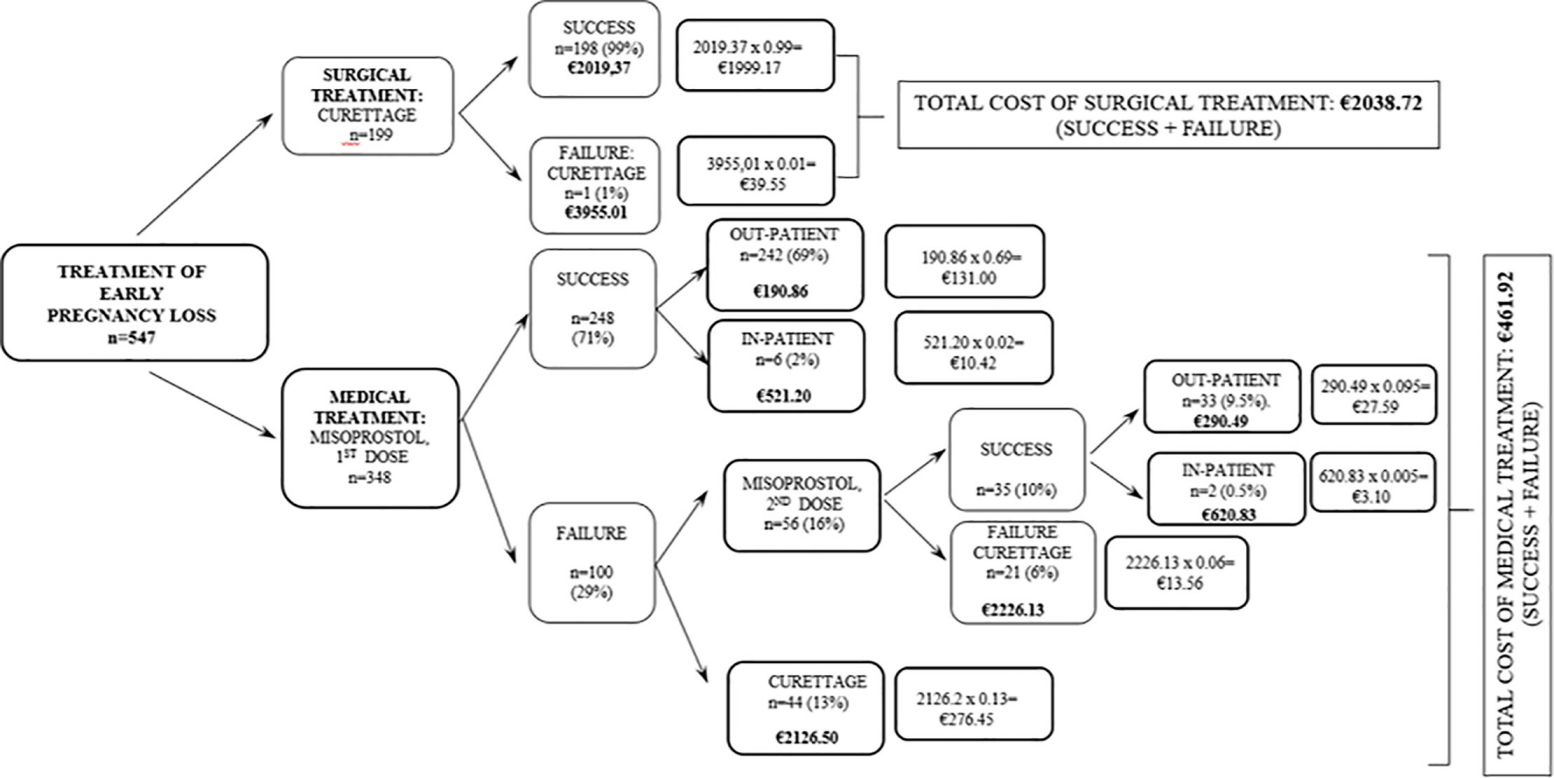
Decision tree for the therapeutic options, with success and failure rates of the patients studied and estimates of the overall medical and surgical treatments (n = 547).

Of the 547 patients who participated in the study, 348 (64%) chose medical treatment and 199 (36%) chose surgical treatment (Fig 1). The baseline characteristics of the groups were similar (Table 1). Although the CRL was slightly longer on average in the surgical treatment group, the difference was not significant.

**Table 1:** Baseline characteristics of patients by treatment received (n = 547).

CHARACTERISTIC	MISOPROSTOL	CURETTAGE
	(n = 348)	(n = 199)
**Age (years)**	34.6±5.4	35.2±5.1
**Previous gestations**	2.2±1.4	2.2±1.3
**Previous miscarriages**	0.6±0.9	0.6±0.9
**Gestational age (weeks)**	9.1±1.6	9.5±1.6
**CRL**	9.7±6.1	11.2±7.1
**TYPE OF PREGNANCY FAILURE**		
Embryonic or fetal death	223 (64%)	149 (75%)
Anembryonic gestation	86 (25%)	28 (14%)
Incomplete abortion	39 (11%)	22 (11%)

*Results expressed as mean (±SD) or number of cases (percentage); CRL: crown-rump length. There were no significant differences between the groups (p>0.05).

The overall effectiveness of the medical treatment was 81% (283/348) (Fig 1). The first dose was 71% effective, while an additional 10% (35 patients) was achieved after the second dose. It should be noted that 44 patients opted for surgical treatment after the failure of the first dose of misoprostol, not trying a second dose. The second dose of the medical treatment failed in 21 patients, who underwent evacuation curettage. Regarding the surgical treatment, the initial effectiveness was 99% since there was one case in which the initial curettage was incomplete and the patient required a second evacuation curettage to complete the treatment, achieving 100% success (Fig 1).

Given that the overall effectiveness of the medical treatment exceeded 80%, the cost minimization study was carried out to compare the two therapeutic alternatives. The costs for each treatment option were calculated (Table 2).

**Table 2:** Costs by procedure and calculation of the total price of each variety of treatment found in the present study.

TREATMENT TYPE	Prices (€)	Total price (€)
**Successful surgical treatment**		
Dilatation & curettage (GRD 381)	2019.37	2019.37
**Failed surgical treatment + re-curettage**		
Dilatation & curettage (GRD 381)	2019.37	
Dilatation & curettage (GRD 381)	2019.37	3955.01
Costs of scheduled consultation +blood group	-83.73	
**Successful medical treatment after first dose**		
Emergency consultation + ultrasound	76.23	
Analytical (preoperative + blood group)	15	
Misoprostol (800 μg)	23.40	190.86
Scheduled consultation (day 7)	76.23	
**In-hospital medical treatment with the first dose**		
Average cost of medical treatment	190.86	
Average cost of extra consultation	76.23	521.20
Average cost from admission from Accident & Emergency	254.11	
**Failed medical treatment after first dose + curettage**		
Average cost of medical treatment	190.86	
Dilatation & curettage (GRD 381)	2019.37	2126.5
Costs of scheduled consultation +blood group	-83.73	
**Successful medical treatment after second dose**		
Average cost of medical treatment	190.86	
Scheduled consultation (day 14)	76.23	290.49
Misoprostol (800 μg)	23.40	
**In-hospital medical treatment with the second dose**		
Average cost of medical treatment (2nd dose)	290.49	
Average cost of extra consultation	76.23	620.83
Average cost from admission from Accident & Emergency	254.11	
**Failed medical treatment after second dose + curettage**		
Average cost of medical treatment (2nd dose)	290.49	
Dilatation & curettage (GRD 381)	2019.37	2226.13
Costs of scheduled consultation +blood group	-83.73	

The cost of a successful surgical treatment was €2019.37 (DRG 381), rising to €3955.01 when the treatment failed and the patient required another curettage. When the medical treatment succeeded with the first dose of misoprostol, the estimated cost was €190,86. If two doses were needed for an effective result, the estimated cost was € 290.49. Six patients had to be admitted to hospital after the first dose of misoprostol for the management of pain or bleeding, although no further action was necessary; in these cases, the cost per patient rose to €521.20. Two patients were admitted after the second dose of misoprostol, with an estimated cost of €620.83. When the medical treatment was not successful and the patient needed curettage, the estimated average cost per patient was €2126.50, if the failure occurred after the first dose of misoprostol (n = 44), and €2226.13 if it happened after the second dose (n = 21).

To assess the overall cost of the two therapeutic approaches, a decision tree with all the possible options was designed and the probability of each event was calculated. The overall cost of each alternative was determined by weighting each partial cost by the estimated probability for each event and then summing them all (Fig 1). With this method, the final cost for medical treatment was estimated as €461.92 compared with €2038.72 for surgical treatment, which represents an estimated average saving of €1576.80 per patient.

## Discussion

The safety, tolerance and acceptability associated with the medical compared with the surgical treatment of abortion has been assessed in several studies, all of which concluded that medical treatment is a safe and tolerable approach, and that the acceptability to patients afterwards is at least equal to that reported after surgical treatment [3,6,7]. We evaluated these three characteristics as part of this study (unpublished data) and found similar results.

Although intuitively it seems clear that the medical treatment of abortion with misoprostol could be a more cost-effective strategy than the surgical one, few published studies have set out to quantify these differences, and none of them was carried out in Spain in the context of the Spanish National Health System [8–11].

The main objective of this study was to compare the costs and effectiveness of both treatments in a non-randomized group of patients (the final decision on the treatment option depended on the patient) with similar characteristics. We intended it to be a cost-minimization study, which meant that it was necessary to test whether the two strategies were equally effective. The threshold of effectiveness by which this equivalence could be judged was based on the study by Zhang et al. [6], which established a minimum effectiveness of 80% for the medical treatment. Our study showed an effectiveness of 81%, similar to the value of 84% achieved by Zhang et al. [6], which means that, in our case, the effectiveness of the two strategies may be considered comparable. However, a review of the literature suggests that the effectiveness of the medical treatment with misoprostol is very variable, with values ranging from 13% [17] to 95% [18]. Although the causes of this variability are not fully understood, it may be related to the wide variety of doses used, the various administration routes (oral, sublingual, vaginal) of the drug [7] as well as the different criteria used to classify the treatment as successful found in the literature. In this study, we used 800 μg of vaginal misoprostol as the initial dose, which is the treatment recommended by SEGO [2] and that used by Zhang et al.[6]. Successful treatment was defined by an endometrial thickness <15 mm, as this is the limit proposed by SEGO [2]. This is also in keeping with other studies [1,19,20], although some other relevant studies consider an endometrial thickness <30 mm as criterion of successful treatment [6]. Nevertheless, no endometrial thickness cut-offs to consider a miscarriage as complete have been validated so far, and endometrial thickness does not even seem to be a predictor of successful treatment [21,22]. To our knowledge, this is the first study that simultaneously compares the clinical efficacy and costs of both methods of treatment for first-trimester spontaneous abortion in Spain.

The method used to study the cost minimization of the medical and surgical treatment options, based on the decision tree method, is similar to that used by Graziosi et al.[11] and Xia et al.[8], but differs as it allows all the clinical variants within the same line of treatment to be visualized. The estimated overall cost of medical treatment in the present study was €461.92, less than a quarter of that of the surgical treatment (€2038.72). These results are consistent with those of Rausch et al. [10], although the latter researchers compared three treatment options (medical, in-patient surgical and out-patient surgical) and found an excess of €284 for the total surgical treatments overall compared with the medical treatment. On the other hand, Xia et al. [8] found no significant differences between the two treatments, although the costs presented by this group were very much lower than the Spanish ones. In China, the estimated cost of evacuation curettage is 175 Yuan (€22.26), which is hardly imaginable in our country [8].

However, our study shows much more striking differences from other studies [3,11]. These differences are mainly due to the inability to break down the total surgical cost of the procedure of DRG 381 into the individual estimates attributable to hospitalisation expenses, anaesthesia, use of the operating theatre and staff expenses, and its result is the estimated average cost, taking into account simple and complex procedures, with and without complications [14]. In addition, the costs of each treatment presented here are closely influenced by specific aspects of the Spanish health model that differ from other models, such as the American [10] (paid for through private insurance) or the Dutch [11] (a public financing and private service provision system). In Spain, this striking difference in costs between the two therapeutic strategies highlights the importance of considering the most cost-efficient alternative when choosing a specific treatment option to optimise the economic resources of the National Health System.

The present study has several limitations and weaknesses. Firstly, this is a quasi-experimental study, not a randomized and blinded one, which reduces its level of evidence. However, the selected groups of patients were completely comparable, so we can assume them to be equivalent regarding statistical inference. Secondly, the <15 mm endometrial thickness limit to define successful treatment is a too strict limit, so it is possible that medical treatment is more effective and therefore more cost-efficient than what our results show. Another limitation is that only the direct costs arising from the pathology to be treated were considered when accounting for the costs of the two types of treatment, whereas the indirect costs, such as those due to days off work, among others, were not included. This would be worth evaluating in subsequent studies.

## Conclusions

In summary, our results show that the medical treatment of first-trimester abortion with misoprostol is a cost-efficient alternative to surgical treatment, with an average saving per patient of more than €1,500. Given its demonstrable safety, effectiveness and tolerance, this therapeutic option should be prioritised above evacuation curettage in patients who meet the treatment criteria.
