# Correction: Medical versus surgical treatment of first trimester spontaneous abortion: A cost-minimization analysis

**DOI:** 10.1371/journal.pone.0214707

**Published:** 2019-03-29

**Authors:** The PLOS ONE Staff

The author initials appear incorrectly in the citation. The correct citation is: Cubo AM, Soto ZM, Haro-Pérez A, Hernández Hernández ME, Doyague MJ, Sayagués JM (2019) Medical versus surgical treatment of first trimester spontaneous abortion: A cost-minimization analysis. PLoS ONE 14(1): e0210449. https://doi.org/10.1371/journal.pone.0210449
